# Fluorescence Manipulation by Gold Nanoparticles: From Complete Quenching to Extensive Enhancement

**DOI:** 10.1186/1477-3155-9-16

**Published:** 2011-05-10

**Authors:** Kyung A Kang, Jianting Wang, Jacek B Jasinski, Samuel Achilefu

**Affiliations:** 1 Chemical Engineering Department, University of Louisville, Louisville, KY 40292, USA; 2 Conn Center, University of Louisville, Louisville, KY 40292, USA; 3 Department of Radiology, Washington University, St. Louis, MO 63105, USA

## Abstract

**Background:**

When a fluorophore is placed in the vicinity of a metal nanoparticle possessing a strong plasmon field, its fluorescence emission may change extensively. Our study is to better understand this phenomenon and predict the extent of quenching and/or enhancement of fluorescence, to beneficially utilize it in molecular sensing/imaging.

**Results:**

Plasmon field intensities on/around gold nanoparticles (GNPs) with various diameters were theoretically computed with respect to the distance from the GNP surface. The field intensity decreased rapidly with the distance from the surface and the rate of decrease was greater for the particle with a smaller diameter. Using the plasmon field strength obtained, the level of fluorescence alternation by the field was theoretically estimated. For experimental studies, 10 nm GNPs were coated with polymer layer(s) of known thicknesses. Cypate, a near infrared fluorophore, was placed on the outermost layer of the polymer coated GNPs, artificially separated from the GNP at known distances, and its fluorescence levels were observed. The fluorescence of Cypate on the particle surface was quenched almost completely and, at approximately 5 nm from the surface, it was enhanced ~17 times. The level decreased thereafter. Theoretically computed fluorescence levels of the Cypate placed at various distances from a 10 nm GNP were compared with the experimental data. The trend of the resulting fluorescence was similar. The experimental results, however, showed greater enhancement than the theoretical estimates, in general. The distance from the GNP surface that showed the maximum enhancement in the experiment was greater than the one theoretically predicted, probably due to the difference in the two systems.

**Conclusions:**

Factors affecting the fluorescence of a fluorophore placed near a GNP are the GNP size, coating material on GNP, wavelengths of the incident light and emitted light and intrinsic quantum yield of the fluorophore. Experimentally, we were able to quench and enhance the fluorescence of Cypate, by changing the distance between the fluorophore and GNP. This ability of artificially controlling fluorescence can be beneficially used in developing contrast agents for highly sensitive and specific optical sensing and imaging.

## Background

Fluorophores have been indispensable optical signal mediators in optical sensing and imaging for a long time and, as an imaging modality, optical imaging has been important because of its higher sensitivity [1]. The signal generation in the fluorphore-mediated sensing is through the excitation of the electrons of the fluorophore by optical energy. The fluorescence emission, therefore, can be altered when the fluorophore is placed near an entity possessing an electromagnetic (plasmon) field. Good candidates for the entity are nano-sized metal particles that form high plasmon field around them, upon receiving optical energy. Exemplary metal entities for this purpose are nanoparticles of gold, silver, platinum, copper, etc. [2,3]. For biological applications, gold is one of only a few appropriate candidates due to its chemical inertness. In addition, the size 'nano' is small enough to incorporate fluorophores or biologicals into it and still able to maintain the resulting product size in a nano-scale. It is, however, large enough to increase their circulation time in blood and the uptake rate by cells, providing a better efficiency in delivery [4,5] in the human body.

When a fluorophore is placed at a relatively short distance, e.g., within 10 nm, from a metal particle possessing a strong plasmon field, the electrons of the flurophore participating in the excitation/emission interact with the field. The interaction results in a change in the fluorescence emission level, i.e., quenching or enhancement. Establishing the relationship between the plasmon field and the resulting fluorescence level can be beneficial in developing highly efficacious optical contrast agents for bio-sensing/imaging. For example, conditional quenching of fluorescence may be effectively used for another form of sensing (i.e., negative sensing or selective quenching) [6]. Enhancement of fluorescence can offer a greater sensitivity and signal-to-noise ratio for molecular sensing/imaging [7,8], especially for the fluorophore with a low quantum yield. If both quenching and enhancement are conditionally implemented in a single fluorophore, then the resulting product can be a highly specific (e.g., FRET or molecular beacon [9]) and highly sensitive optical contrast agent.

In terms of the scientific progress in manipulating the fluorescence of fluorophores by metal nanoparticles, the quenching phenomena [9-12] appeared to be studied separately from the enhancement [13-25]. Lately, more researchers are recognizing both quenching and enhancement of fluorescence caused by the metal nanoparticles [26,27]. A few research groups have performed excellent theoretical analyses with experimental verification [3,28-30]. Not all researchers used the same approach in their analyses but they appeared to agree that there are two main factors affecting the changes on the fluorescence by metal nanoparticles: (1) the plasmon field generated around the particle, by the incident light, increases the excitation decay rate of the fluorophore, which in turn, enhances the level of fluorescence emission; and (2) the dipole energy around the nanoparticle reduces the ratio of the radiative to non-radiative decay rate and the quantum yield of the fluorophore, resulting in the fluorescence quenching.

We have theoretically studied the changes in the excitation decay rate and the quantum yield of a fluorophore that are caused by the plasmon field on/around a GNP. Fluorescence levels of a near infrared (NIR) fluorophore Cypate placed at various distances from the GNP surface were experimentally measured and compared with those obtained by the theoretical study.

We hope that our study results will be helpful for improving the performance of the fluorescence contrast agents.

## Theoretical Analysis on Fluorescene Quenching and Enhancement by Metal Nanoparticles

**Figure 1:**
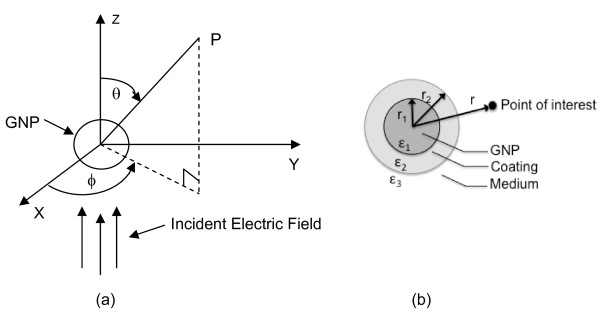
**Theoretical system**. (a) The coordinates used in the computation of the plasmon field strength on and around a GNP and (b) a schematic diagram of a GNP with polymer coating in a medium.

The change in the fluorescence of a fluorophore placed near a metal nanoparticle is caused by the plasmon field generated by the particle, and the nature and level of the change depend upon the field strength. The field strength on and around a metal nanoparticle upon the exposure to incident light depends on the metal type, particle size, surface modification of the particle, and wavelength of the incident light. Several mathematical models are currently available for computing the plasmon field strength on and around metal nanoparticles [28-31], relating the parameters listed above. We have selected a model developed by Neeves and Birnboim [31] because it fits well for the particles used for biomedical studies, e.g., polymer coated particle in water medium. This model calculates the plasmon field strength considering only dipolar contribution. The system is assumed to have a particle concentration dilute enough to neglect the inter-particle interaction and its intrinsic dielectric non-linearity may be neglected [31]. The model uses a spherical coordinate system (Figure 1). The plasmon field strength (E_p_) at a position r, generated by an incident light (E_o_) by a metal particle (radius, r_1_) coated with a shell (thickness, r_2_-r_1_), in a surrounding medium, can be described as in Eq. 1. For our study, we are assuming that the system has an azimuthal symmetry [].(1)

And the field strength inside the shell (E_p_^layer^; in our case, biopolymer coating) is:(2)

where  and  are unit vectors in *r *and *θ *in the spherical coordinates, respectively, and(3)(4)(5)

ε_1_, ε_2_, ε_3_, and ε_ο _are the dielectric permittivity values of the particle, the shell, the outer suspending medium, and vacuum, respectively. In our study, the metal nanoparticle is GNP and the fluorophore is Cypate. Cypate was separated from the GNP surface by a polymer shell of a known thickness. For a GNP, ε_1 _is wavelength dependent and may be described by the Drude-Lorentz model (Eq. 6) [31].(6)

where *i *denotes imaginary number; ω, the frequency of the incident light; ω_o_, bound electron resonant frequency; and ω_p_, plasma frequency.(7)

where τ_f _and τ_b _are the free electron scattering time and bound electron decay time, respectively. V_f _is the Fermi velocity and τ_o_, the free electron scattering time in the bulk material. Note that, for the particles without the shell, r_1 _is r_2._

Most parameter values used for our system are from Neeves and Birnboim [31] and they are: ω_o _= 7.0 × 10^15 ^sec^-1^; ω_p _= 1.3 × 10^16 ^sec^-1^; V_f _= 1.38 × 10^6 ^m/sec; τ_o _= 9.3 fsec; τ_b _= 0.2 fsec; and ε_o _= 8.85 × 10^-12 ^C^2^/N m^2^. ε_2 _and ε_3 _are usually constant. For our experimental system, the shell was a bi-layer coating of poly(allylamine hydrochloride) (PAH) and poly(sodium-4-styrene sulfonate) (PSS) and its ε_2 _value is 2.5 ε_o _[32]. Our medium was water and ε_3 _for water (the medium) is 1.76 ε_o_. The plasmon field strength around a particle changes with the direction from the particle surface, and, in our analysis, the field strength at θ = 0 (the parallel direction to the incident light) is used.

The normalized enhancement of the excitation decay rate  has the relationship with the normalized plasmon field strength (or) as in Eq. 8 [28,32,33].(8)

where the superscript '^o^' is for the value of the system without GNP.

The quantum yield (q) indirectly influenced by the plasmon field E_p _[29] can be described as:(9)

where *γ*_r _is the radiative decay rate, *γ*_abs _is the additional non-radiative decay rate resulted from the radiated energy absorbed by the particle, and q^o ^is the intrinsic quantum yield of the fluorophore. For a spherical particle with a quasi-static polarizability,  (Eq. 8). Because the second term represents the energy absorption by the particle, if the wavelength of the emission peak is very close to that of the particle resonance peak (usually at around 520 nm for GNPs), this term has a very significant contribution to the quantum yield change. The intrinsic quantum yield (q^o^) also has an important role, if it is very small.

The normalized absorption rate is expressed by Eq. 10 [29].(10)

where ω_em _is the frequency of the emission light; k,; c, the speed of light; , the transition dipole moment; and x, y, z are the axes in the Cartesian coordinates on the particle surface. It should be noted that, in our study, we analyzed the values in the z direction only, and for this condition, p_x _= p_y _= 0.

The fluorescence enhancement rate (Φ) is, therefore, the combined effect of the enhancement of the excitation decay rate and the change in the quantum yield, both influenced by the plasmon field.(11)

## Materials and methods

### 1. Materials and Instruments

10 nm GNP colloids were purchased from Ted Pella (Redding, CA). The mean diameter of the particle is 10.0 nm with a coefficient of variation <10%, according to the manufacturer. Poly(allylamine hydrochloride) (PAH; MW = 15,000) and poly(sodium-4-styrene sulfonate) (PSS; MW = 13,500) were from Sigma Aldrich (St. Louis, MO) and Polymer Standard Service (Mainz, Germany), respectively. Cypate [34-36] was produced by the Achilefu group. 1-Ethyl-3-(3-dimethylaminopropyl) carbodiimide hydrochloride (EDC) was from Thermo Scientific (Rockford, IL). Potassium cyanide (KCN) was from Fluka (St. Louis, MO).

Sonication was performed using 200 Ultrasonic Cleaner (Branson; Danbury, CT) and 550 Sonic Dismembrator (Fisher scientific; Pittsburgh, PA). Centrifugation was performed using Eppendorf 5415 R Centrifuge (Eppendorf AG; Hamburg, Germany). The dialysis cassette (MW cut-off, 20,000) used to purify the product was from Thermo Scientific (Rockford, IL).

For the absorption studies, DU^®^520 UV-VIS spectrophotometer (Beckman; Fullerton, CA) was used and the fluorescence of Cypate was measured in 96-well Uniplates (Whatman; Florham Park, NJ) using Spectra Gemini XPS fluorometer (Molecular Devices Corp.; Sunnyvale, CA). Computer simulations were performed using MATLAB R2008a (The Mathworks Inc., Natick, MA). For analyzing various particles produced, we used a dynamic light scattering (DLS) particle size analyzer (90Plus/BI-MAS; Brookhaven Instruments Co.; Holtsville, NY) and a transmission electron microscope (TEM; Tecnai™ HR FEG-TEM; FEI co.; Hillsboro, Oregon)

### 2. Methods

Coating GNPs with the bi-polymer PAH/PSS was performed following the procedure of Schneider *et al*. [12]. We, however, used commercially available 10 nm GNPs, while they made their own GNPs at a size of 13 nm. Before the coating, 10 nm GNPs in citric acid were centrifuged at 7,000 rpm for 3 hrs to remove excess citric acid. After discarding the supernatant, GNPs were dispersed in DI water and the bi-polymer was coated on the GNPs.

Cypate was placed on the polymer-coated GNPs in the form of Cypate conjugated PAH. Conjugation of Cypate was performed as follows: To avoid self-quenching of Cypate fluorescence by crowding, the amount of Cypate was limited to be approximately 1% of available amine groups in PAH. 4.1 mg of Cypate (6.5 μmol) was dissolved in 10 mL DI water and the solution was added drop-wise to 20 mL of the solution containing 100 mg PAH. 2 mg of coupling agent EDC (10.4 μmol) was added and the solution was stirred in dark for 12 hrs. To remove un-reacted Cypate and EDC, the solution was dialyzed using a dialysis cassette in 2 L DI water, covered with aluminum foil to avoid bleaching, for 12 hrs, and then it was dried under vacuum overnight. The resulting, green solid was then dispersed in 1.5 mL DI water and the solution was centrifuged at 11,000 rpm for 45 min to ensure no un-reacted Cypate in the final product. This step was repeated twice. The resulting product, Cypate-conjugated PAH (PAH-Cy), was a green solid and the yield of Cypate was estimated to be 86%, based on the Cypate absorption at 780 nm.

To coat PAH-Cy onto the outermost layer of GNP-(PAH/PSS)_i_, first, PAH-Cy was dissolved in DI water and the amount of Cypate in the solution was adjusted to 30 μM (absorbance 0.495 at 780 nm). The subscript '*i*' is the number of (PAH/PSS) bi-layers on the GNP surface, and it ranges from 0-3 in our study. The solution was sonicated for 2 min, and then stirred for 2 hrs. To this solution, the aqueous solution of GNP-(PAH/PSS)_i _at 12.7 nM was added drop-wise and stirred for 12 hrs. The GNP-(PAH/PSS)_i_-(PAH-Cy) was then purified by centrifuging at 11,000 rpm for 45 min and the pellet was re-dispersed in DI water. This step was repeated. To all (PAH-Cy)-coated GNPs, a layer of PSS was added according to the above procedure to yield GNP-(PAH/PSS)_i_-(PAH-Cy)/PSS.

To dissolve the gold core from GNP-(PAH/PSS)_i_-(PAH-Cy)/PSS by KCN, we followed the procedures described by Schneider, et al. [12]. The fluorescence levels of the samples were monitored until little fluorescence change was noted. After the measurements were completed, the absorption spectra of the samples were measured to study the presence/absence of GNPs and the potential change in PAH-Cy spectra after the process.

## Results and Discussion

Our system for the study, as previously described, is a PAH-Cy layer coated on the PAH/PSS layer(s) that was placed on the surface of a 10 nm GNP, We selected gold because it is chemically inert and non-toxic, and its surface can be easily modified for adding other molecules, such as, fluorophores and targeting biomolecules. Cypate is a derivative of an FDA approved fluorophore Indocyanine Green (ICG). ICG has been extensively used as a chromophore and also as a fluorophore in clinical practices [37]. Both ICG and Cypate have excitation and emission peaks at 780 nm and 830 nm, respectively. These peaks are in NIR region, avoiding the naturally occurring fluorescence in the tissue and also allowing deep penetration into tissues. The quantum yields of ICG are 0.0028 in water and 0.012 in blood or human serum albumin solution [38].

An important issue on using nanoparticles for biomedical purpose is, assuming that the metal is not cytotoxic, the effect of their size on the long term toxicity in excretory organs and on the cell uptake rate. Although more studies are required to fully understand the issue, the general sense on the appropriate size (including surface modification) should be in the range of 10-100 nm, with a neutral surface charge [4]. Most metal nanoparticles used for biomedical purpose are coated with bio-compatible, hydrophilic polymer layers on their surface. Also, various bio-molecules including disease targeting molecules and/or drugs are often incorporated in/on the layer. The core GNP size of our interest is, therefore, less than 50 nm, assuming that the maximum thickness for the GNP surface modification is less than 25 nm and, for this study, we selected the GNP core size to be between 5 and 30 nm.

It should be noted that the values presented in the figures are relative values and, the value one (1) is the same as the value of our control, the system without GNPs.

### 1. Theoretical Studies

**Figure 2:**
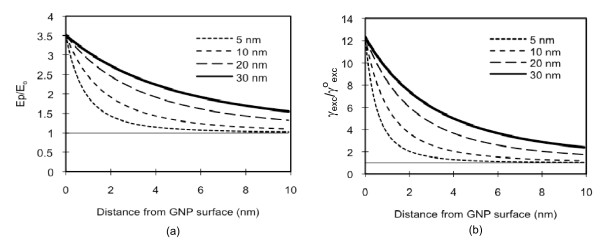
**Theoretical estimation of GNP plasmon field strength and excitation decay rate generated at 780 nm**. (a) The normalized plasmon field distribution and (b) the normalized enhancement of the excitation decay rate, with respect to the distance from the surface of the GNP at a size of 5, 10, 20 or 30 nm, when the incident light at 780 nm is applied.

For a predetermined incident light wavelength, the plasmon field distribution around a GNP depends upon the GNP size (Eq. 1-8). Figure 2a illustrates the normalized, plasmon field distributions on and around GNPs of sizes of 5, 10, 20 or 30 nm, for the incident light wavelength at 780 nm, the excitation peak for Cypate. For all sizes, the field strengths on the particle surface are similar. The field strength decreases rapidly with the increase in the distance from the surface, as also shown by other researchers [28-30]. For smaller particles, the strength decreases faster and, thus, at the same distance from the GNP surface, it is weaker. If greater field strength is desired at a particular distance from a GNP, then larger GNPs need to be used, and *vice versa*. The normalized, enhancement of excitation decay rate (, Figure 2b), which is the main cause for fluorescence enhancement, shows more significant differences with the size, because it has a relationship with the square of the field strength (Eq. 8). In sum, one can achieve a desired enhancement in the decay rate by appropriately selecting the GNP size and the distance from the GNP.

**Figure 3:**
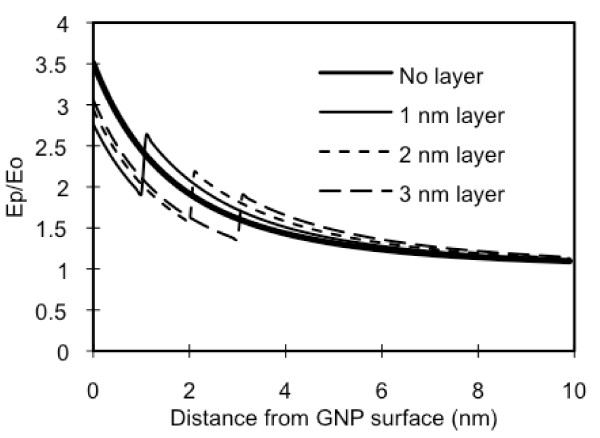
**Effect of polymer coating on plasmon field strength**. The normalized plasmon field distributions on/around a 10 nm GNP coated with PAH/PSS bi-layer(s), at thicknesses of 0, 1, 2, and 3 nm, when the incident light at 780 nm is applied.

The polymer on the GNP may also affect the plasmon field distribution. Figure 3 illustrates the field distribution on and around a 10 nm GNP with a PAH/PSS coating [ε_2 _= 2.5 ε_ο_] at a thickness of 0, 1, 2 or 3 nm. For all cases, within the polymer layers, the field strengths are lower than the ones without. If one intends to place fluorophores inside the polymer layer then one should be aware that the plasmon field strength at the same distance from the GNP with a polymer layer is significantly lower than that of a bare GNP. The field strength immediately outside the coating is slightly higher than that of a bare GNP but the difference is minor. It should be noted that some metal shells can increase the field strength [31].

**Figure 4:**
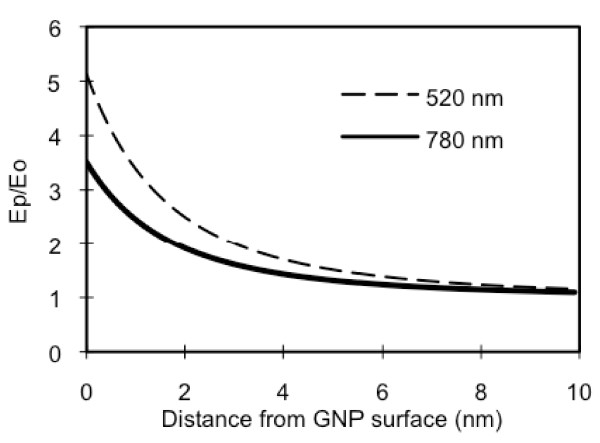
**Effect of incident light wavelength on plasmon field strength**. Theoretical estimations of the normalized, plasmon field distribution on/around a 10 nm GNP, for an incident light wavelengths at 520 and 780 nm.

GNPs at a size range of our interest usually absorb light the strongest at around 520 nm, the plasma resonance peak. The field distribution at the resonance peak was computed and compared with the value at the excitation peak (780 nm) of Cypate, for a 10 nm GNP (Figure 4). As expected, on the particle surface, the field strength generated by 520 nm is approximately 1.4 times of that by 780 nm, and therefore, the enhancement in the excitation decay rate at 520 nm should be more than twice if that at 780 nm. However, if the fluorophore has an emission peak close to 520 nm, *q *will also be decreased significantly due to the large second term value in the denominator of Eq. 9. In other words, at 520 nm, the enhancement in the resulting fluorescence may not occur. Instead, quenching may dominate.

**Figure 5:**
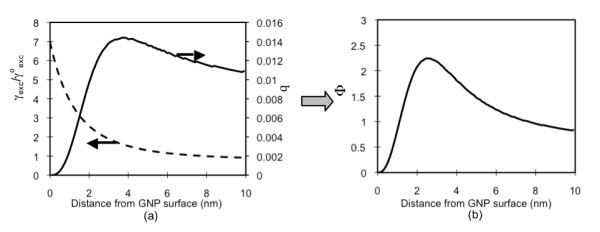
**Cypate fluorescence alternation by GNP**. (a) The normalized, enhancement of the excitation decay rate () and the Cypate quantum yield (*q*), affected by the plasmon field generated by a 10 nm GNP in water, upon the receipt of light at 780 nm; (b) the resulting enhancement rate of Cypate fluorescence. (GNP is coated with PAH/PSS)

**Figure 6:**
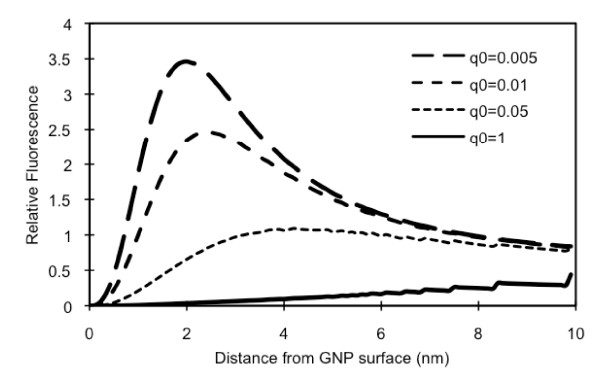
**The effect of the intrinsic quantum yield on the fluorescence of Cypate with the distance from a 10 nm GNP. (GNP is coated with PAH/PSS)**.

Figure 5 shows the enhancement of excitation decay rate (; Figure 5a; dotted lines) and the change in the Cypate quantum yield (q; Figure 5a; solid lines) influenced by the plasmon field generated by a 10 nm GNP, when the incident light at the wavelength 780 nm is applied. In our experiment, GNPs are coated with PAH/PSS bi-layer(s) and a PAH-Cy layer was placed on the bi-layer(s), and therefore, in the plasmon field strength computation, we included a shell of the bi-layer(s). For this computation, the intrinsic quantum yield value used for Cypate was 0.012. On the GNP surface, the value is as high as 7 times of that without a GNP, but the Cypate *q *value is zero (0). The emission wavelength of Cypate (830 nm) is far from the GNP resonance wavelength (520 nm) and therefore, the second term of the denominator of Eq. 9 is not significant except on or very close to the GNP surface. Cypate, however, has a very low intrinsic quantum yield (q^o ^= 0.012), and therefore, the third term of the denominator in Eq. 9 becomes significant. As shown in Eq. 10 the enhancement in the resulting emission decay rate (Φ) is by the combined effect of  and . Figure 5b shows that on the surface of the GNP, no fluorescence is emitted but at around 3 nm from the surface the emission rate is enhanced approximately 2.5 times. To simply illustrate the effect of q^o ^on the fluorescence, we artificially varied the q^o ^value of Cypate, while all other parameters/conditions remain the same (Figure 6). Here, q^o ^was varied in the range of 0.01-1. As q^o ^increases, the enhancement level decreases. For the ones with q^o ^greater than 0.05, the enhancement does not occur at a distance within 10 nm from a 10 nm GNP. In other words, for increasing fluorescence with GNPs, fluorophores with low quantum yields have more potential. The distance providing the highest fluorescence increases slightly with the increase in q^o^.

**Figure 7:**
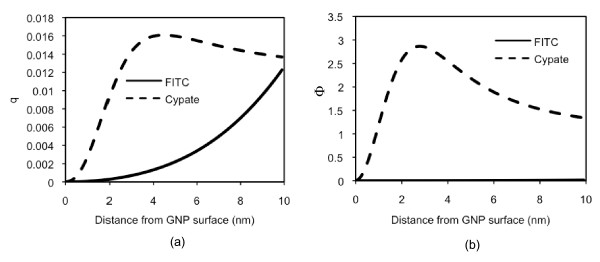
**Changes in FITC quantum yield and fluorescence by GNP, compared to those of Cypate**. (a) Quantum yields (*q*) of FITC and Cypate when the light is applied at the peak of their respective excitation wavelengths (Ex/Em; 495/521 nm and 780/830 nm, respectively) to a 10 nm GNP and (b) the resulting fluorescence. The FITC fluorescence is extremely low in the range of distance studied.

To illustrate the effects of the wavelength and the quantum yield together on the resulting fluorescence, we have selected a fluorophore with properties very different from those of Cypate (Figure 7). Fluorescein isothiocyanate (FITC) has the excitation and emission peaks at 495 and 521 nm (at around GNP resonance peak), respectively, and its intrinsic quantum yield is 0.93 [39], approximately 100 times that of Cypate's. Figure 7a shows the quantum yield of Cypate and FITC, influenced by a 10 nm GNP. The quantum yield of FITC becomes lower than that of Cypate at the distance up to 10 nm from the GNP. As high as the enhancement of the excitation decay rate at around 520 nm (Figure 4), the resulting fluorescence of FITC (Figure 7b) still shows significant quenching (little to no fluorescence) in the entire range, due to the high emission light absorption by GNP (the second term in the denominator of Eq. 9).

### 2. Experimental studies

**Figure 8:**
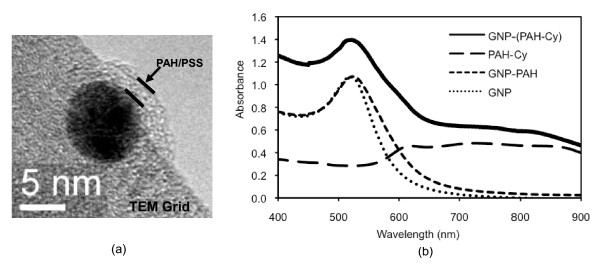
**Characterization of nanoparticle products**. (a) TEM image of a polymer coated 10 nm GNP, and (b) Absorption spectra of GNP, GNP-PAH, PAH-Cy, and GNP-(PAH-Cy).

To experimentally test the effect of the plasmon field strength generated by a GNP on the resulting fluorescence of a fluorophore, it is necessary to separate a fluorophore from a GNP by a known distance. This can be done by coating GNPs with a polymer layer of known thickness and placing the fluorophore outside the polymer layer. We have used a method developed by Schneider et al. [12], i.e., placing two polymers with opposite charges, i.e., poly(allylamine hydrochloride; PAH) and poly(sodium-4-styrene sulfonate; PSS), on GNPs. PAH is an amine-rich, cationic polymer and PSS is anionic and these two form a strong and stable bi-layer structure. By adding predetermined numbers of the polymer layers on GNPs, one can vary the thickness of the polymer-shell on GNPs and the shell thickness becomes the distance that separates Cypate molecules from the GNP surface. We were able to add up to three layers on 10 nm GNPs [GNP-(PAH/PSS)_0-3_] without difficulties, where the subscripts 0-3 denotes the number of the layer. For more than three layers, it was more difficult to keep the dispersity of the resulting nanoparticles for a long time. The thickness of the first PAH/PSS composite layer produced by Schneider, et al. [12] was 1.5 ± 0.3 nm. Polymer imaging by TEM is usually difficult due to the poor response of polymers to the electron beam. In our study, we tried to place the polymer coated GNP at the edge of TEM grid so that we could achieve a better contrast. The average thickness of one bi-layer was estimated to be approximately 2 nm with a standard deviation of 0.5 nm (Figure 8a). We also tried the DLS method but the values were less consistent than those by TEM, and, therefore, we decided to use the TEM values.

Cypate was then placed outside the PAH/PSS layer(s), in the form of Cypate-conjugated PAH (PAH-Cy), as described in the Method section. Two carboxyl groups of Cypate can be covalently conjugated to the amine groups of PAH (PSS does not have amine groups). As stated in the Method section, to avoid a potential self-quenching of Cypate fluorescence by crowding, the amount of Cypate used was only approximately 1% of available amine groups in PAH. The thickness of single PAH layer alone was assumed to be 1 nm (as a half of the PAH/PSS by-layer). For all particles with PAH-Cy layer, an additional layer of PSS was placed to protect the Cypate layer. The distance between Cypate and a GNP surface was assumed to be the thickness of (PAH/PSS)_i _layer(s) plus a half thickness of the PAH layer. For example, for the PAH-Cy applied on the first layer of PAH/PSS, the thickness was assumed to be 2.5 nm. In addition, to observe the fluorescence of Cypate on the GNP surface, Cypate was adsorbed onto the GNP surface directly.

The fluorescence levels generated by PAH-Cy before and after the conjugation to GNPs should be compared with the same amount of PAH-Cy and the quantification of Cypate would have to be done by the absorption property of Cypate. To confirm that there was no significant changes in the absorption property of PAH-Cy optical characterization of PAH-Cy, PAH coated GNP, and PAH-Cy conjugated GNP [i.e., GNP-(PAH-Cy)] was performed. As can be seen in Figure 8b, the GNP absorption spectrum has a distinctive peak at 520 nm. PAH coated GNPs also have a peak at around 520 nm but slightly red-shifted, as was in the study by Schneider et al. [12]. PAH-Cy has signature absorption between 700 and 880 nm. GNP-(PAH-Cy) shows the combined absorption of the GNP-PAH and PAH-Cy, indicating that the optical properties of PAH-Cy were not significantly affected by the conjugation process to GNPs.

Next, the relationship between the layer thickness on the GNP surface and the fluorescence of PAH-Cy was studied. For all samples, the Cypate concentration was adjusted to 30 μM. Then, the fluorescence intensities of GNP-Cypate, GNP-(PAH-Cy) and GNP-(PAH/PSS)_0-3_-(PAH-Cy) were measured at the excitation and emission wavelengths of 780 and 830 nm, respectively.

**Figure 9:**
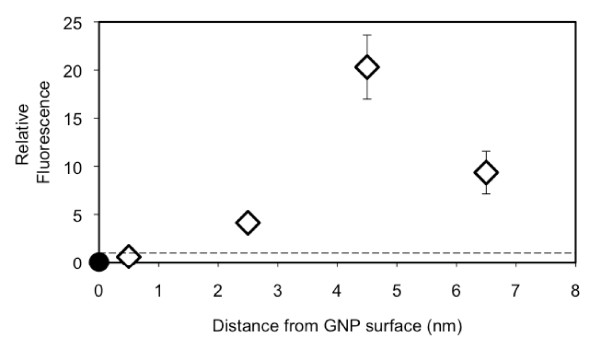
**Relative Cypate fluorescence with change in the distance from the GNP surface**. The distance was varied by varying numbers of the (PAH/PSS) bi-layer on the GNP. The dotted line indicates the fluorescence of PAH-Cy as a control.

**Figure 10:**
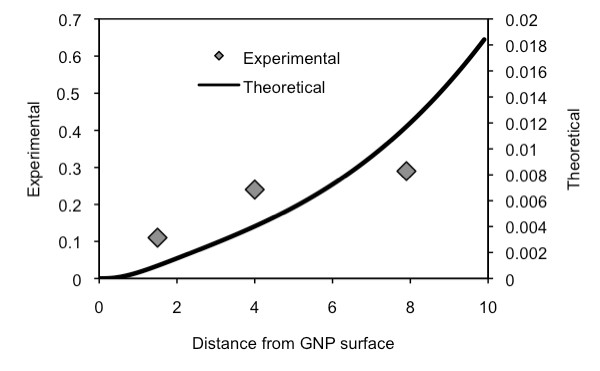
**Comparison of experimental and theoretical results of FITC fluorescence**. Experimental data is from Schneider, et al. (PAH-FITC on PAH/PSS layers on 13 nm GNP) [12].

Figure 9 illustrates the fluorescence level with respect to the distance (i.e., polymer layer thickness) between the GNP surface and Cypate, in a normalized form with the fluorescence of PAH-Cy as a control. It should be noted that, since each bi-layer has a thickness of 2 nm, the interval in x-axis is 2 nm. The sample of the Cypate bound directly onto the GNP surface showed complete quenching. The level of quenching decreased as the fluorophore moved from the surface to approximately 1 nm from it [i.e., GNP-(PHA-Cy)]. When the distance became 2.5 nm, the fluorescence became 5 times of the control and at 4.5 nm, the fluorescence was enhanced as much as 17 times. At 6.5 nm, the enhancement decreased but still more than 10 times of the control. It is expected that, eventually, the fluorescence would approach to its control level as the thickness increases further. This result confirms that fluorescence of a fluorophore can be quenched and also enhanced by a GNP. In the case of Schneider, et al. [12], the fluorescence was quenched for all thicknesses they studied. The main reason for this is because of the difference in the properties of the fluorophores (i.e., FITC v.s. Cypate), as also shown in Figure 7b. The theoretical prediction of the fluorescence for Schneider's system and their experimental results are shown in Figure 10. The trend of the two results is similar, although experimental values show then intenstities approximately 30 times higher than the theoretical ones.

**Figure 11:**
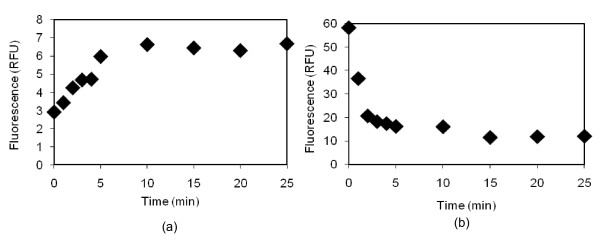
**Verification of GNP effect on fluorescence alteration**. The changes in the fluorescence of samples of (a) GNP-(PAH-Cy) and (b) GNP-(PAH/PSS)_2_-(PAH-Cy), as KCN was added and the gold core was dissolved.

To verify whether the fluorescence alteration was, in fact, caused by GNP, we removed the source of the alteration by dissolving the gold from our nanoparticle products using potassium cyanide (KCN) [12]. We then observed the changes in fluorescence during the process of the GNP removal (Figure 11). The polymer shell and the fluorophore layer were expected to remain unchanged during and after gold was dissolved [12]. For this study, we selected the particles with the polymer layer showing the most quenching, i.e., GNP-(PAH-Cy), and the ones with the most enhancement, i.e., GNP-(PAH/PSS)_2_-(PAH-Cy). As can be seen in Figure 11a, for GNP-(PAHCy), the fluorescence was restored as the GNP was dissolved. For GNP-(PAH/PSS)_2_-(PAHCy), fluorescence enhancement slowly disappeared with the removal of gold (Figure 11b). After the fluorescence measurements were completed, the absorption spectra of the samples were taken to ensure a complete GNP removal and to see the potential changes in the PAH-Cy absorption spectrum. The PAH-Cy did not change its absorption spectrum with the addition of KCN while the absorption peak at 520 nm (GNP signature peak) disappeared for all samples (data not shown). This study result again confirms that GNPs can both quench and enhance the fluorescence of a fluorophore, and that, for a particular GNP size, the level of quenching and enhancement depends upon the distance between the GNP and the fluorophore.

**Figure 12:**
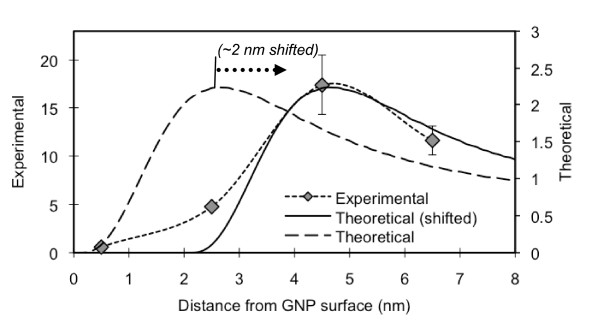
**Comparison of the experimental and theoretical results of the Cypate fluorescence by 10 nm GNPs**. Experimental data show an enhancement level of 7-8 times of the theoretical estimation. The distance from the GNP surface displaying the maximum fluorescence is approximately 2 nm longer than that for the result of the theoretical system of a single GNP and a Cypate molecule.

**Figure 13:**
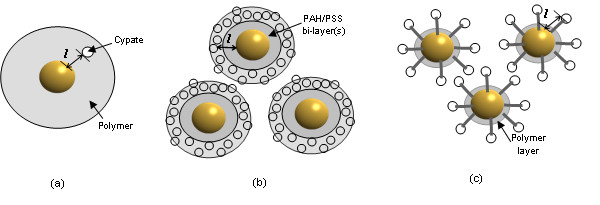
**Systems of the GNP and Cypate molecule in the study**. (a) The system for the theoretical analysis: A Cypate molecule is placed at a distance of *l *from a GNP; (b) The system used in the experiment, A PAH-Cy layer placed on PAH/PSS bi-layer(s) of thickness of *l*, coated GNP; and (c) Cypate placed via spacers on a GNP coated with biocompatible polymer.

Next, we plotted the theoretical (Figure 5b) and experimental (Figure 9) results in one figure (Figure 12) and compared the two. The general trend of the two appeared to be similar. However, the distance for the maximum fluorescence in the experimental data appeared to be approximately 2 nm longer than the one theoretically estimated. The level of enhancement for the experimental system was approximately 7-8 times greater than the theoretical results, as was reported by Schneider, et al. (Figure 10) [12]. The discrepancies may be due to the differences in the theoretical and experimental systems: The theoretical system was based on a single GNP and a single Cypate molecule (Figure 13a) inside PAH/PSS bi-layer, while, in the experimental system, multiple Cypate molecules inside a PAH layer (~1 nm thick) was placed onto the PAH/PSS layer(s) on GNPs (Figure 13b). Although the concentrations of Cypate and the GNPs in the samples were set to be low to minimize the inter-fluorophore and inter-particle interactions, the experimental system was more complicated than the system used in the theory development and these interactions might still exist. Nevertheless, the theoretical prediction can provide an approximate length scale for the quenching and enhancement for a desired design. Because the theoretical model is much simpler than the actual system to be used, a thorough experimental verification should be followed to produce the desired products. The fluorophore/GNP configuration used for most optical contrast agent development may be represented by Figure 13c. According to our experiences, with this design, the maximum enhancement levels for the experimental and theoretical results were similar (data not presented here).

**Figure 14:**
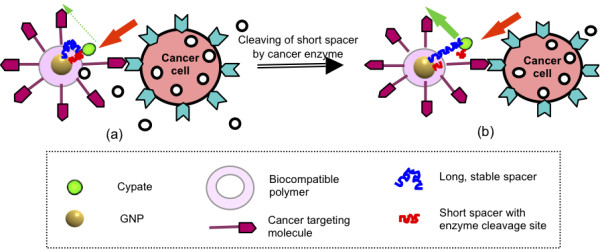
**Design of a highly specific and sensitive optical contrast agent using GNP**. (a) The cancer targeting molecule (red arrow) reacts with the receptor on the cancer cell; (b) The short spacer is cleaved by a cancer enzyme (**o**) and then the distance between Cypate and the GNP assumes the length of the long spacer, resulting in enhanced fluorescence.

Fully taking advantage of this unique phenomenon, we are currently developing a novel, fluorescing nano-entity that can be effectively used for cancer detection and diagnosis. The design of this entity is a Cypate conjugated to GNP via two spacers, one short and one long (Figure 14). The short spacer must be sufficiently short to ensure that the Cypate fluorescence is quenched. In addition, the short spacer includes a moiety that can be cleaved by an enzyme (**o**) secreted by the target cancer cell. The long spacer should be biocompatible and biochemically stable. Its length should be such that the Cypate fluorescence is maximally enhanced. The GNP also includes a cancer targeting biomolecule (red arrow), as well as being coated with a biocompatible, hydrophilic polymer layer [in our case, a combination of a hydrocarbon chain and a short polyethylene glycol (PEG)] chain. Ideally, after administering the entity to a patient and prior to finding the cancer, the entity emits little or no fluorescence because the short spacer ensures Cypate to be within the distance for fluorescence quenching by the GNP. Once it arrives at the cancer site, the targeting molecule reacts with a receptor on the cancer cells (Figure 14a) and the short spacer is cleaved by the cancer secreting enzyme. This results in an increase in the distance between Cypate and the GNP to the length of the long spacer. When excitation light is applied, the fluorescence of Cypate, consequently, is emitted at a highly enhanced level (Figure 14b).

## Conclusions

Surface plasmon field of metal nanoparticles, especially of GNPs, may be used for artificially manipulating fluorescence. This tool of fluorescence manipulation can be highly beneficial for optical, molecular sensing and imaging. To better understand the plasmon field distribution on and around a GNP and its effect on the fluorescence changes, we have theoretically studied the plasmon field intensity on/around GNPs with various properties. The field intensity is highest on the GNP surface and decreases rapidly with the distance from the surface for all sizes, and the rate of decrease is greater for the smaller size.

In the process of designing the quenching and enhancement effect by metal nanoparticles, the main factors to be considered are: (1) the metal type of the particle in case metals other than gold are used. The dielectric permittivity of the metal determines the plasmon field distribution; (2) the nanoparticle size, i.e., field strength and the enhancement of the excitation decay rate depends on the particle size; (3) the wavelength (or fluorophore) to be used. The field strength depends upon the excitation wavelength, and the level of absorption of the emission light by the nanoparticle varies depending upon the emission wavelength; (4) the intrinsic quantum yield of the flurophore: It is one of the major factors that determine the quantum yield of the fluorophore placed near the nanoparticle; (5) the placement of a shell on the surface. The plasmon field distribution may change significantly depending on the material properties of the shell on the particle.

In designing effective optical contrast agents, using both theoretical and experimental results on the relationship between the fluorescence of a fluorophore and the plasmon field strength (or in a more practical sense, the distance between the fluoropohore and a GNP at a known size) will be highly beneficial. For the researchers who plan to use this concept in practice, it is suggested that they first theoretically estimate the plasmon field distribution on/around the metal nanoparticle for a particular set of the system with a particular set of parameters (e.g., the metal type and the size of the particle and the excitation and emission wavelengths and the intrinsic quantum yield of the fluorophore and the type and thickness of the shell). Once they have a set of experimental relationships for a particular system, they may be able to predict the relationships for another system using the theory, instead of performing many sets of experiments.

We have also performed experimental studies on fluorescence changes of Cypate, an NIR fluorophore, by placing it at various distances from a GNP. For the studies, 10 nm GNPs were coated with polymer layers at known thicknesses and a Cypate layer was placed outside the polymer layers. The level of Cypate fluorescence was then correlated with the distance from the GNP surface (i.e., polymer layer thickness). Fluorescence became almost completely quenched on the particle surface and approximately 17 times stronger than without GNP, at ~4.5 nm from the GNP surface. As the distance increased further, the enhancement decreased. The results of this study confirm that the plasmon field both quenches and enhances the fluorescence and the effect is strongly dependent on the distance from the particle surface (i.e., field strength).

After a thorough theoretical analysis and experimental verification on the relationship between the fluorescence of a fluorophore and a GNP, one can use the relationship to produce novel optical contrast agents with high sensitivity and specificity.

## Summary Points

• Fluorescence of a fluorophore can be artificially altered by metal nanoparticles. Factors that can be manipulated to obtain a desired fluorescence are: the metal type and size of the nanoparticle, and distance between the fluorophore and the particle.

• The quenching or enhancement of the fluorescence of a fluorophore highly depends upon the excitation and emission wavelengths of the fluorophore.

• Theoretical prediction of the fluorescence may be beneficially used, at an initial stage of development, in designing a fluorophore-nanoparticle contrast agent, with thorough experimental verification in a later stage.

• Enhancing fluorescence of FDA approved, NIR fluorophores by GNPs may be highly beneficial for medical imaging.

## List of Abbreviations

Cy: Cypate; DLS: dynamic light scattering; Em: emission; Ex: excitation; FITC: fluorescein isothiocyanate; FRET: fluorescence resonance energy transfer; GNP: gold nanoparticle; ICG: Indocyanine Green; NIR: near infrared; PAH: poly(allylamine hydrochloride); PAH-Cy: Cypate conjugated poly(allylamine hydrochloride); PAH/PSS: poly(allylamine hydrochloride)/poly(sodium-4-styrene sulfonate) bi-layer; PSS: poly(sodium-4-styrene sulfonate); q: quantum yield; q^o^: intrinsic quantum yield; TEM: transmission electron microscopy

## Declaration of Competing interests

A patent application was filed with the content of this article, through the University of Louisville. There is no other competing interest.

## Authors' contributions

KAK conducted the research design and completed the manuscript, JW conducted the theoretical studies, JBK provided consultation on the mathematical model and obtained TEM images, and SA developed the fluorophore Cypate. All authors read and approved final manuscript.
